# Effects of Vitamin D and Dexamethasone on Lymphocyte Proportions and Their Associations With Serum Concentrations of 25-Hydroxyvitamin D_3_ *In Vitro* in Patients With Multiple Sclerosis or Neuromyelitis Optica Spectrum Disorder

**DOI:** 10.3389/fimmu.2021.677041

**Published:** 2021-07-29

**Authors:** Eun Bin Cho, Jong Hwa Shin, Soonwook Kwon, Juhyeon Kim, Jin Myoung Seok, Byoung Joon Kim, Ju-Hong Min

**Affiliations:** ^1^Department of Neurology, Gyeongsang Institute of Health Science, Gyeongsang National University, College of Medicine, Jinju, South Korea; ^2^Department of Neurology, Gyeongsang National University Changwon Hospital, Changwon, South Korea; ^3^Department of Neurology, Samsung Medical Center, Sungkyunkwan University School of Medicine, Seoul, South Korea; ^4^Department of Neurology, Neuroscience Center, Samsung Medical Center, Seoul, South Korea; ^5^Department of Neurology, Inha University School of Medicine, Incheon, South Korea; ^6^Department of Neurology, Gyeongsang National University Hospital, Jinju, South Korea; ^7^Department of Neurology, Soonchunhyang University Cheonan Hospital, Soonchunhyang University College of Medicine, Cheonan, South Korea; ^8^Department of Health Sciences and Technology, Samsung Advanced Institute for Health Sciences & Technology (SAIHST), Sungkyunkwan University, Seoul, South Korea

**Keywords:** vitamin D, lymphocytes, memory B cells, *CYP24A1*, multiple sclerosis, neuromyelitis optica spectrum disorder

## Abstract

**Background:**

Clear associations have been found between vitamin D deficiency and several autoimmune diseases including multiple sclerosis (MS). However, the benefits of vitamin D supplementation on disease management remain a matter of debate.

**Objective and Methods:**

Patients with MS (*N*=12) and neuromyelitis optica spectrum disorder (NMOSD; *N*=12) were enrolled along with 15 healthy controls. Changes in lymphocyte subset proportions during stimulation of their peripheral blood mononuclear cells (PBMCs) with the active form of vitamin D, 1,25-dihydroxyvitamin D_3_ (1,25(OH)_2_D_3_), and correlations with serum concentrations of the vitamin D precursor 25-hydroxyvitamin D_3_ (serum 25(OH)D_3_) were explored. The impact of 1,25(OH)_2_D_3_ stimulation on the expression of vitamin-D-responsive genes in immune cells was also investigated.

**Results:**

In both MS and NMOSD, stimulation of PBMCs with 1,25(OH)_2_D_3_ followed by steroid suppressed the proliferation of total lymphocytes and T cells. The ratio of CD19^+^CD27^+^ memory B cells (Bmem) to all B cells after stimulation with 1,25(OH)_2_D_3_ was negatively correlated with serum 25(OH)D_3_ in MS (Spearman’s *ρ*=–0.594, *p*=0.042), but positively correlated in NMOSD (Pearson’s *r* = 0.739, *p*=0.006). However, there was no relationship between the ratio of Bmem to CD19^+^CD24^+^CD38^+^ regulatory B cells and serum 25(OH)D_3_ in either MS or NMOSD. In addition, the level of 1,25(OH)_2_D_3_-induced *CYP24A1* mRNA expression in PBMCs was significantly and negatively correlated with serum 25(OH)D_3_ (for ΔC_T_, *r*=0.744, *p*=0.014) in MS.

**Conclusion:**

These findings suggest a beneficial impact of stimulation of PBMCs with vitamin D followed by steroid on the T-cell population. The association between patient serum 25(OH)D_3_ and the proportion of Bmem under immune-cell stimulation differed between MS and NMOSD. Further investigations are warranted with larger patient populations.

## Introduction

1,25-Dihydroxyvitamin D_3_ (1,25(OH)_2_D_3_), the active form of vitamin D, is receiving increasing attention due to its role as a regulator of the immune system (1). Immune cells, such as macrophages, dendritic cells, and activated lymphocytes, express both the vitamin D receptor (VDR) and 1α-hydroxylase (CYP27B1), the key enzyme that catalyzes the bioactivation of 1,25(OH)_2_D_3_ from its precursor 25-hydroxyvitamin D_3_ (25(OH)D_3_) (2–4). Vitamin D suppresses the production of proinflammatory cytokines such as interferon-γ, interleukin (IL)-2, and IL-17, enhances the secretion of anti-inflammatory cytokines such as IL-4 and IL-10, and shifts the balance toward immune responses mediated by T helper (Th) type 2 cells and regulatory T cells (3, 5). In addition, vitamin D interferes with B-cell proliferation and differentiation into memory B cells (Bmem) and antibody-secreting plasma cells (6, 7). From this perspective, the large amount of data linking decreased vitamin D levels to an abnormal immune response, such as increased autoimmunity, is of great concern (8–13). Low vitamin D levels have been correlated with disease activity or disability in various autoimmune disorders, including systemic lupus erythematosus (SLE), Sjögren’s syndrome, multiple sclerosis (MS), and neuromyelitis optica spectrum disorder (NMOSD) (8, 9, 11, 12).

MS and NMOSD are autoimmune inflammatory demyelinating diseases that affect the central nervous system (CNS). Autoreactive T cells in the periphery and T cell–B cell collaboration contribute to the pathogenesis of CNS autoimmunity. CNS antigen-specific T cells, specifically CD4^+^ Th1 cells and Th17 cells, and antibodies against the aquaporin-4 water channel (AQP4) are believed to play key roles in the development of MS and NMOSD, respectively (14, 15). In the periphery, B cells, and especially Bmem, may serve as antigen-presenting cells, and activate and differentiate the autoreactive T cells into the Th17 lineage by producing cytokines such as IL-6 and IL-21 (14, 16). In addition, Th17 and T follicular helper cells produce the cytokines IL-17a and IL-6, which promote granulocyte activation, and B-cell differentiation and antibody production (17). Vitamin D may exert an immunomodulating effect by suppressing the inflammatory autoimmune response. However, the benefit of vitamin D in terms of therapeutic applications is not clear for either MS or NMOSD (11, 18, 19).

This study investigated the effects of vitamin D on immune cells through peripheral blood mononuclear cell (PBMC) stimulation in patients with MS or NMOSD, and in healthy controls (HCs). The associations between serum 25(OH)D_3_ concentration (hereafter serum 25(OH)D_3_) and the proportions of lymphocyte subsets following stimulation with 1,25(OH)_2_D_3_ were also evaluated, and 1,25(OH)_2_D_3_-induced changes in mRNA expression of the genes encoding VDR (*VDR*), the vitamin-D-activating and vitamin-D-degrading enzymes 1α-hydroxylase (*CYP27B1*) and 24-hydroxylase (*CYP24A1*), respectively, and IL-10 (*IL-10*) were explored.

## Methods

### Subjects

Patients with MS (20) (*N*=12) and AQP4-antibody-positive NMOSD (15) (*N*=12) were enrolled at Samsung Medical Center in Seoul, South Korea between November 2016 and August 2018. A total of 20 ml of peripheral venous blood samples were taken during remission in all patients. The same amount of venous blood was also obtained from 15 HCs who did not have a history of acute or chronic disease and had not been taking any medication during the previous 3 months. Total serum 25(OH)D_3_ was measured using commercially available enzyme-linked immunosorbent assay kits (Eagle Biosciences, NH, USA) according to the manufacturer’s instructions. The study was approved by the Institutional Review Board of Samsung Medical Center and written informed consents were obtained from all subjects.

### PBMC Preparation and Culture

Whole blood was collected into lithium heparin tubes and PBMCs were then separated using density-gradient centrifugation on Ficoll-Paque PLUS (GE Healthcare Biosciences, Pittsburgh, PA, USA). The isolated PBMCs were suspended in fetal bovine serum (FBS; Life Technologies, Grand Island, NY, USA) and 10% dimethyl sulfoxide (Sigma Aldrich, St. Louis, MO, USA) at a concentration of 1×10^7^ cells/ml, and then stored in liquid nitrogen until required for the stimulation experiments.

The PBMCs were cultured with RPMI (Roswell Park Memorial Institute) 1640 medium containing l-glutamine supplemented with 10% certified inactivated FBS and 50 units of penicillin/streptomycin (Life Technologies). They were then incubated with a nonspecific stimulation mixture of 10 ng/ml lipopolysaccharide (LPS; Sigma ldrich), 100 ng/ml CD40 ligand (CD40L; Enzo Biochem, New York, NY, USA), and 5 nM cytosine-phosphate-guanosine-oligodeoxynucleotides (CpG-ODN 2006; Invivogen, USA), either alone (LPS+CD40L+CpG-ODN) or in combination with steroid (dexamethasone, 10 nM, Sigma Aldrich), 1,25(OH)_2_D_3_ (1 μM, Sigma Aldrich), or both steroid and 1,25(OH)_2_D_3_. When used, 1,25(OH)_2_D_3_ was added 24 hours prior to LPS+CD40L+CpG-ODN. The cells were incubated for 72 hours, after which they were analyzed with flow cytometry.

### Immunophenotyping by Flow Cytometry

The percentages of total lymphocytes and T cells, B cells, regulatory B cells (Breg), and Bmem in the PBMC samples were determined by flow cytometric analysis of the surface markers CD3, CD19, CD24, CD38, and CD27. In this study, CD19^+^CD24^+^CD38^+^ cells were defined as Breg and CD19^+^CD27^+^ cells as Bmem (21). PBMCs were incubated with the following surface marker antibodies (all from BD Biosciences, San Jose, CA, USA) for 30 min at 4°C in the dark: anti-CD19-APC-Cy7 (clone SJ25C1), anti-CD3-PerCPCy5.5 (clone SK7), anti-CD24-FITC (Clone ML5), anti-CD38-PE (clone HIT), anti-CD27-PE (clone M-T271). They were then washed twice with phosphate-buffered saline (PBS), suspended in PBS, and analyzed using FACS Canto II flow cytometry with FACS DIVA software (version 6.1.3, BD Biosciences).

### Real-Time Polymerase Chain Reaction

Total RNA was isolated using the RNeasy Mini kit (Qiagen, Valencia, CA, USA) according to the manufacturer’s instructions. cDNA was synthesized by reverse transcription at 37°C for 30 min, followed by reverse transcriptase (RT) inactivation at 95°C for 5 min using the Fast Advanced RT buffer and Enzyme mix (Thermo Fisher Scientific, Waltham, MA, USA). Each gene-expression assay consisted of a 6-carboxy-fluorescein (FAM)-dye-labeled TaqMan MGB (minor groove binder) probe and two polymerase chain reaction (PCR) primers (TaqMan human FAM assays). The target genes were *VDR* (Hs00172113_m1), *CYP24A1* (Hs00167999_m1), *CYP27B1* (Hs00168017_m1), *IL-10* (Hs00961622_m1), and glyceraldehyde-3-phosphate dehydrogenase (*GAPDH*; Hs02786624_g1). All of these genes were obtained from Thermo Fisher Scientific. *GAPDH*, which is present in all genomes, is commonly used as an endogenous control (i.e., a housekeeping gene) for analyzing the relative levels of gene expression. Real-time PCR was carried out using the TaqMan Fast advanced Master Mix (Thermo Fisher Scientific). Uracil *N*-glycosylase (UNG) was used to prevent the possible reamplification of carryover PCR products. The thermal cycle conditions were 50°C for 2 min (AMPerase UNG activation), 95°C for 20 sec (Taq activation), and then PCR for 40 cycles of 95°C for 1 sec and 60°C for 20 sec. Relative expression levels of DNA were calculated automatically using QuantStudio 6 Pro Real-Time PCR System software (Thermo Fisher Scientific). Cycle threshold (C_T_) values were defined as the thresholds required for reference amplification. The average C_T_ was calculated for each target gene (C_T, target gene_) and for *GAPDH* (C_T,_ *_GAPDH_*); the difference (ΔC_T_) was calculated for each target gene using the equation ΔC_T_ = C_T, target gene_−C_T,_ *_GAPDH_*.

### Statistical Analysis

Lymphocyte subset percentages in PBMC samples were compared between the MS, NMOSD, and HC groups using one-way analysis of variance (ANOVA) or Kruskal–Wallis tests. Two-way ANOVA or Friedman’s test was used to compare the proportions of specific lymphocytes and ΔC_T_ values for *VDR*, *CYP24A1*, *CYP27B1*, and *IL-10* mRNA expression among the four stimulation groups (LPS+CD40L+CpG-ODN alone or in combination with 1,25(OH)_2_D_3_, steroid, or both) for each of the three study groups. Bonferroni’s correction for multiple comparisons was performed. Correlations between the study participants’ serum 25(OH)D_3_ and the percentage of each lymphocyte subset or ΔC_T_ values for mRNA expression were evaluated using Pearson’s correlation or Spearman’s correlation. SPSS (version 20, SPSS, Chicago, IL, USA) and Prism (version 8.4.3, GraphPad, La Jolla, CA, USA) were used for statistical analysis and data presentation. The criterion for statistical significance was *p*<0.05.

## Results

The characteristics of the study participants are summarized by group in **Table 1**. Patients with NMOSD were significantly older than HCs (mean age, 44 *vs.* 32 years; *p*=0.013). Vitamin D supplementation was reported by 25% (3/12) of the MS patients and 33% (4/12) of the NMOSD patients, but none of the HCs. Serum 25(OH)D_3_ was higher in the NMOSD group than in their HC counterparts (*p*=0.030).

**Table 1:** Demographics and serum concentrations of 25(OH)D_3_ in study subjects.

	HCs (*N* = 15)	MS (*N* = 12)	NMOSD (*N* = 12)	*p* value
Age	32.3 ± 5.2	36.8 ± 9.8	44.1 ± 14.2	0.016^a^
Female, *N* (%)	8 (53.3)	9 (75.0)	11 (91.7)	0.085
Disease duration, months	N/A	27.5 (4.55–1.75)	53.5 (21.75–115.75)	0.236
25(OH)D_3_, mg/dl	18.1 ± 5.8	20.9 ± 11.7	31.4 ± 18.5	0.028^a^
On taking 1,25(OH)_2_D_3_, *N* (%)	0	3 (25.0)	4 (33.3)	0.060
ARR	N/A	1.0 (0.638–4.0)	0.7 (0.4–1.1)	0.148
EDSS	N/A	1.0 (0.625–2.875)	3.0 (1.25–3.0)	0.078
Use of drugs, *N* (%)	N/A	9 (75.0)^b^	11(91.7)^c^	0.590

N, number; ARR, annualized relapse rate; EDSS, Expanded Disability Status Scale; HCs, healthy controls; MS, multiple sclerosis; NMOSD, neuromyelitis optica spectrum disorder; Values are presented as either mean ± SD or median (IQR) unless otherwise indicated.

a significant only between HCs and NMOSD.

b interferon β-1b (n=4), interferon β-1a (n=4), and teriflunomide (n=1).

c azathioprine (n=5), mycophenolate mofetil (n=3), hydroxychloroquine (n=2), and methotrexate (n=1).

**Figure 1:**
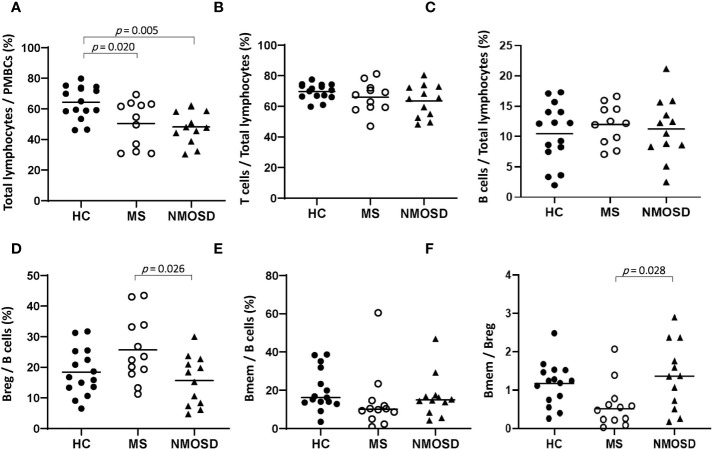
The proportions of lymphocytes in PBMC samples from HCs and patients with MS or NMOSD. The percentage of total lymphocytes was lower in MS and NMOSD than in HCs. The Breg/B cell ratio was higher and the Bmem/Breg ratio was lower in patients with MS than in those with NMOSD. The data are presented as scatter plots in which the horizontal line indicates the mean **(A–D)** or median **(E, F)**. Only statistically significant *p* values (*p*<0.05) are presented. Bmem, CD19^+^CD27^+^ memory B cell; Breg, CD19^+^CD24^+^CD38^+^ regulatory B cell; HC, healthy control; MS, multiple sclerosis; NMOSD, neuromyelitis optica spectrum disorder; PBMC, peripheral blood mononuclear cell.

In PBMC samples, the proportion of total lymphocytes was significantly lower in both the MS and NMOSD groups than in the HC group (*p*=0.020 and 0.005, respectively). The frequency of Breg among B cells was lower and the Bmem/Breg ratio was significantly higher in the NMOSD group than in the MS group (*p*=0.026 and 0.028, respectively); there were no significant differences in these parameters between the MS and HC groups or between the NMOSD and HC groups (**Figure 1**). There were no differences in the percentages of T cells, B cells, and Bmem between any of the study groups (HC *vs.* MS *vs.* NMOSD).

### Changes in the Proportion of Lymphocytes in Response to Stimulation With 1,25(OH)_2_D_3_

Nonspecific stimulation with LPS+CD40L+CpG-ODN resulted in a significant increase in total lymphocyte percentage (*p*=0.014) and significant reductions in the Bmem percentage and Bmem/Breg ratio (*p*=0.010 and 0.028, respectively) in the NMOSD group, while the T-cell percentage decreased in the MS group (*p*=0.017) (**Supplementary Figure 1**).

**Figure 2:**
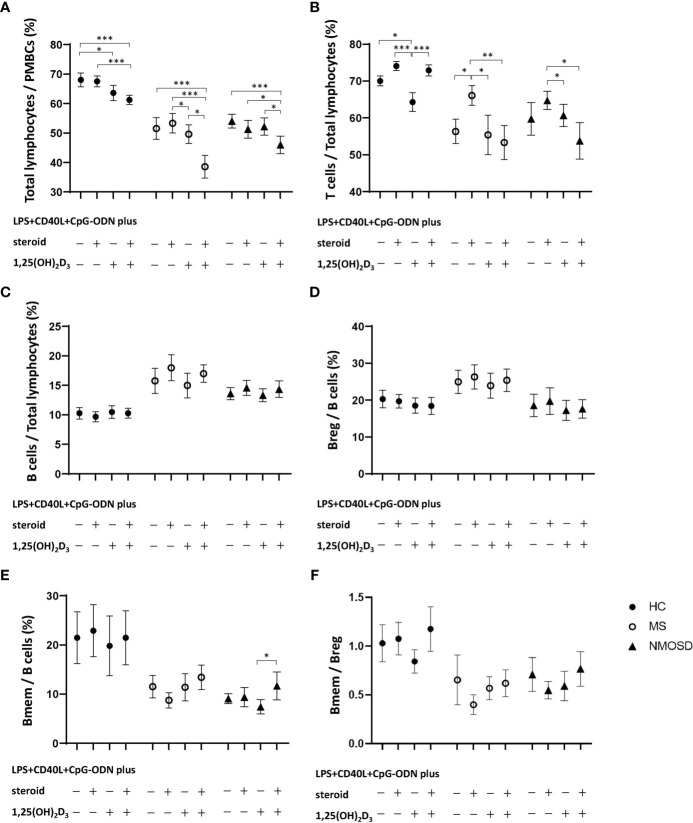
Changes in the proportion of lymphocytes in PBMC samples in response to nonspecific stimulation with LPS+CD40L+CpG-ODN with or without 1,25(OH)_2_D_3_, and/or steroid (dexamethasone) in HCs and in patients with MS or NMOSD. Stimulation with 1,25(OH)_2_D_3_ significantly reduced the percentages of **(A)** total lymphocytes and **(B)** T cells in HCs. However, stimulation with 1,25(OH)_2_D_3_ followed by steroid caused significant decreases in the percentages of **(A)** total lymphocytes and **(B)** T cells in the MS and NMOSD groups compared with those achieved by stimulation with steroid treatment alone. 1,25(OH)_2_D_3_ stimulation did not alter the proportions of **(C)** B cells or **(D)** Breg, or **(F)** the Bmem/Breg ratio in any of the three study groups. Stimulation with 1,25(OH)_2_D_3_ followed by steroid significantly increased the proportion of **(E)** Bmem in PBMC samples compared with 1,25(OH)_2_D_3_ stimulation alone in the NMOSD group. Data are mean and standard-error values. Statistically significant differences are indicated by horizontal bars: ^*^ *p*<0.05, ^**^ *p*<0.01, ^***^ *p*<0.001. 1,25(OH)_2_D_3_, 1,25-dihydroxyvitamin D_3_; Bmem, CD19^+^CD27^+^ memory B cell; Breg, CD19^+^CD24^+^CD38^+^ regulatory B cell; CD40L, CD40 ligand; CpG-ODN, cytosine phosphate guanosine oligodeoxynucleotides; HC, healthy control; LPS, lipopolysaccharide; MS, multiple sclerosis; NMOSD, neuromyelitis optica spectrum disorder; PBMC, peripheral blood mononuclear cell.

Stimulation of HC PBMCs with 1,25(OH)_2_D_3_ significantly reduced the percentage of total lymphocytes (*p*=0.027), regardless of subsequent stimulation with steroid (**Figure 2A**). 1,25(OH)_2_D_3_ also caused a reduction in the percentage of total lymphocytes in PBMCs from the MS and NMOSD groups, but only when steroid was added after 1,25(OH)_2_D_3_ (*p*<0.05 *vs.* steroid or 1,25(OH)_2_D_3_ alone for both patient groups).

The proportion of T cells among total lymphocytes was also significantly reduced by stimulation of HC PBMCs with 1,25(OH)_2_D_3_ (*p*=0.012), but that proportion increased upon subsequent stimulation with steroid. In the MS and NMOSD groups, the percentage of T cells increased after PBMC stimulation with steroid (*p*=0.012 and 0.158, respectively), but not if they were first stimulated with 1,25(OH)_2_D_3_ (**Figure 2B**). 1,25(OH)_2_D_3_ stimulation did not alter the proportions of B cells or Breg, or the Bmem/Breg ratio in any of the three study groups (**Figures 2C, D, F**). However, stimulation with 1,25(OH)_2_D_3_ followed by steroid significantly increased the proportion of Bmem in PBMC samples compared with 1,25(OH)_2_D_3_ stimulation alone in the NMOSD group (**Figure 2E**).

### Associations Between Serum 25(OH)D_3_ and Percentage of Bmem

**Figure 3:**
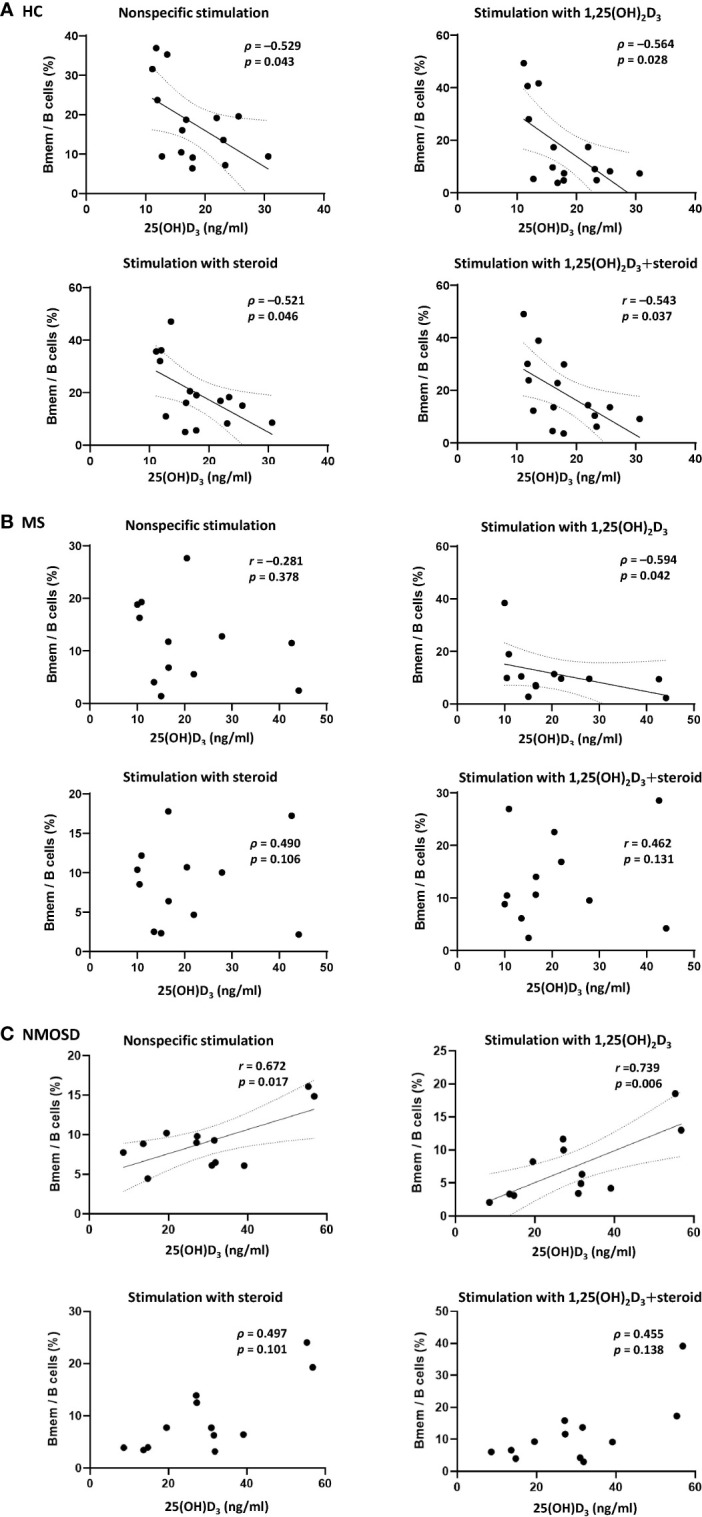
Correlations between the proportion of Bmem in samples of PBMCs and serum 25(OH)D_3_ in HCs and in patients with MS or NMOSD. A negative correlation between the percentage of Bmem and serum 25(OH)D_3_ was found **(A)** in HCs regardless of the type of stimulation (i.e., steroid or 1,25(OH)_2_D_3_), and **(B)** in patients with MS only after stimulation of PBMCs with 1,25(OH)_2_D_3_. **(C)** In NMOSD, significant positive correlations were found after PBMC stimulation, with or without 1,25(OH)_2_D_3_. Linear regression lines indicate significant correlations. 1,25(OH)_2_D_3_, 1,25-dihydroxyvitamin D_3_; Bmem, CD19^+^CD27^+^ memory B cell; HCs, healthy controls; MS, multiple sclerosis; NMOSD, neuromyelitis optica spectrum disorder; serum 25(OH)D_3_, serum concentration of 1,25-dihydroxyvitamin D_3_.

Prior to PBMC stimulation, there were no correlations between the Bmem/B cell ratio and serum 25(OH)D_3_ in any of the study groups. However, after nonspecific PBMC stimulation with LPS+CD40L+CpG-ODN, there was a negative correlation between the Bmem/B cell ratio and serum 25(OH)D_3_ in the HC group (Spearman’s *ρ*=0.529, *p*=0.043), and a positive correlation (Pearson’s *r* = 0.731, *p*=0.007) in the NMOSD group (**Figures 3A, C**). Additional stimulation with 1,25(OH)_2_D_3_ did not alter these correlations for either group. In the MS group, an association between Bmem/B cell ratio and serum 25(OH)D_3_ was found only following stimulation with 1,25(OH)_2_D_3_, which yielded a negative correlation (*ρ*=–0.594, *p*=0.042; **Figure 3B**). There was no correlation between the Breg/B cell ratio in PBMC samples and serum 25(OH)D_3_ in any of the groups, regardless of the stimulation conditions. A negative association was found between the Bmem/Breg ratio and serum 25(OH)D_3_ after stimulation of PBMCs with 1,25(OH)_2_D_3_ only in the HC group (*ρ*=–0.583, *p*=0.023).

### Changes in Expression Levels of *VDR, CYP27B1, CYP24A1*, and *IL-10* Genes in Response to 1,25(OH)_2_D_3_

**Figure 4:**
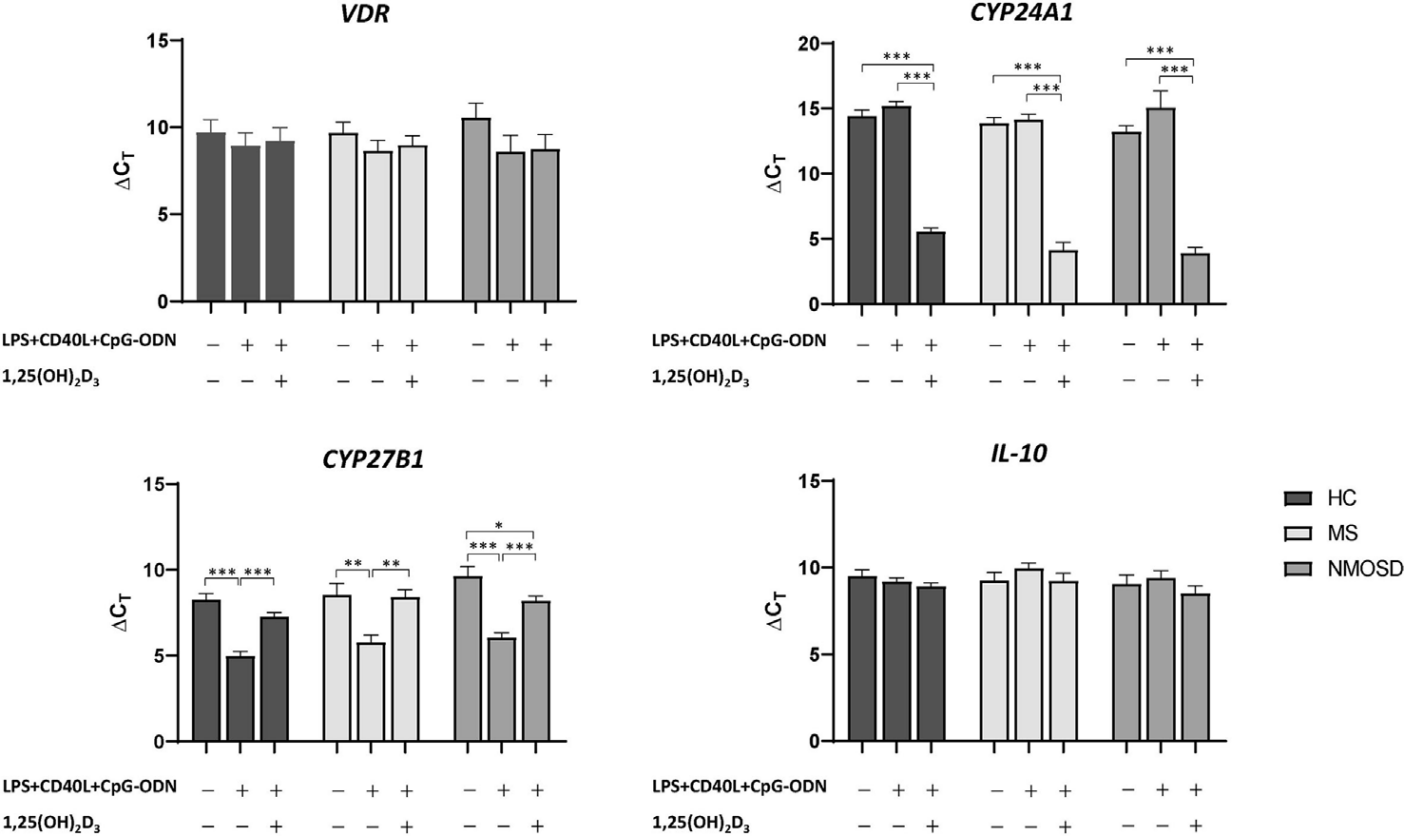
Changes in the mRNA expression of *VDR*, *CYP27B1*, *CYP24A1*, and *IL-10* in PBMCs in response to stimulation with LPS+CD40L+CpG-ODN (nonspecific) with or without 1,25(OH)_2_D_3_. In HCs and patients with MS or NMOSD, stimulation with 1,25(OH)_2_D_3_ increased the mRNA expression of *CYP24A1* and decreased that of *CYP27B1* over those observed in samples stimulated with LPS+CD40L+CpG-ODN alone. ^*^ *p*<0.05, ^**^ *p*<0.01, ^***^ *p*<0.001. 1,25(OH)_2_D_3_, 1,25-dihydroxyvitamin D_3_; CD40L, CD40 ligand; CpG-ODN, cytosine phosphate guanosine oligodeoxynucleotides; HC, healthy control; LPS, lipopolysaccharide; MS, multiple sclerosis; NMOSD, neuromyelitis optica spectrum disorder; PBMC, peripheral blood mononuclear cell.

1,25(OH)_2_D_3_-induced changes in the PBMC mRNA expression levels of *VDR*, *IL-10*, *CYP27B1*, and *CYP24A1* are shown in **Figure 4**. PBMCs from 15 HCs, 11 MS patients, and 13 NMOSD patients were included (**Supplementary Table 1**). PBMC stimulation with LPS+CD40L+CpG-ODN increased *CYP27B1* mRNA expression, but the addition of 1,25(OH)_2_D_3_ suppressed it. PBMC *CYP24A1* expression was also induced by stimulation with 1,25(OH)_2_D_3_. These findings were observed in PBMCs from all study groups and were statistically significant. However, the expression levels of *VDR* and *IL-10* mRNA were unaffected by stimulation of PBMCs, with or without 1,25(OH)_2_D_3_.

**Figure 5:**
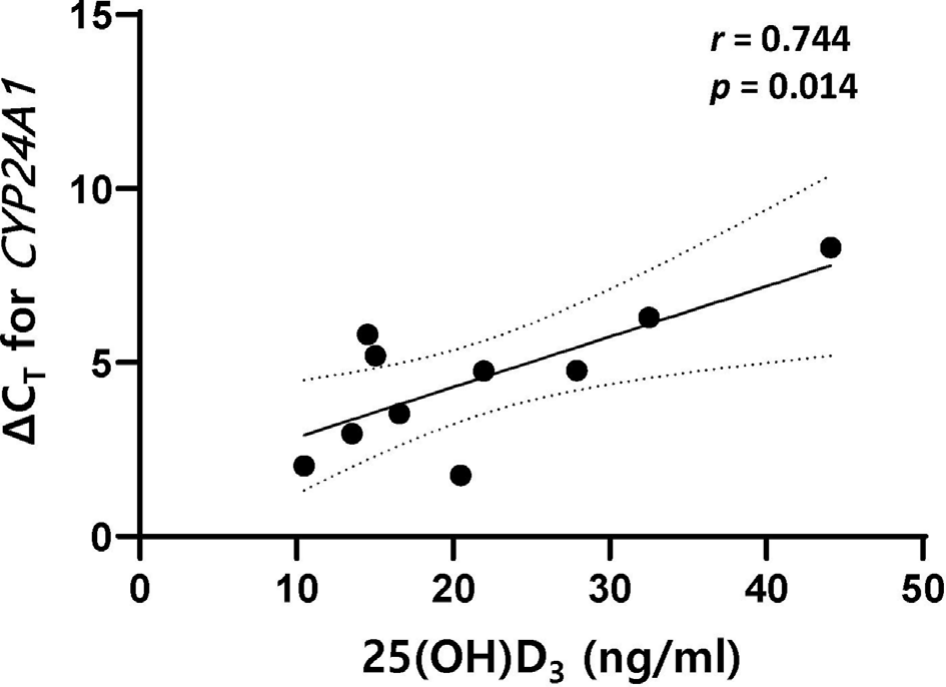
There was a positive correlation between ΔC_T_ for *CYP24A1* and serum 25(OH)D_3_ in patients with MS. The mRNA expression level of *CYP24A1* after stimulation with 1,25(OH)_2_D_3_ was lower in MS patients with higher serum 25(OH)D_3_. 1,25(OH)_2_D_3_, 1,25-dihydroxyvitamin D_3_; ΔC_T_, difference in cycle threshold between that for the target gene and that for *GAPDH*; MS, multiple sclerosis; serum 25(OH)D_3_, serum concentration of 1,25-dihydroxyvitamin D_3_.

In patients with MS, the level of 1,25(OH)_2_D_3_-induced *CYP24A1* mRNA expression in PBMCs was significantly and negatively correlated with serum 25(OH)D_3_ (for ΔC_T_, *r*=0.744, *p*=0.014); however, no significant association was found for *VDR*, *CYP27B1*, and *IL-10* mRNA expression (**Figure 5**). In HCs, the level of 1,25(OH)_2_D_3_-induced *IL-10* mRNA expression was positively correlated with serum 25(OH)D_3_ (for ΔC_T_, *r*=0.590, *p*=0.026). In patients with NMOSD, there was no correlation between mRNA expression of *VDR*, *CYP27B1*, *CYP24A1*, or *IL-10* and serum 25(OH)D_3_, irrespective of stimulation with 1,25(OH)_2_D_3_.

## Discussion

The findings of this study demonstrate that 1,25(OH)_2_D_3_ exerted an inhibitory effect on the proliferation of lymphocytes, and especially T cells. However, in patients with MS and NMOSD, this 1,25(OH)_2_D_3_-induced suppression of lymphocytes (total and T cells) was obvious upon subsequent stimulation with steroid. In addition, statistically significant associations in the opposite direction were found between serum 25(OH)D_3_ and the Bmem/B cell ratio after PBMC stimulation in patients with MS and NMOSD; it should be noted that there was no correlation between the Bmem/Breg ratio and serum 25(OH)D_3_. Moreover, the expression of *CYP24A1*, the gene encoding a 1,25(OH)_2_D_3_-catabolizing enzyme, was less expressed in response to 1,25(OH)_2_D_3_ in MS patients with higher serum 25(OH)D_3_.

Stimulation with 1,25(OH)_2_D_3_ followed by steroid decreased the percentage of total lymphocytes and T cells in PBMCs from patients with MS and NMOSD. This finding could be explained by the known immune regulatory role of vitamin D. The active form of vitamin D, 1,25(OH)_2_D_3_, induces monocyte proliferation, which contributes to the innate immune response and attenuates the cytotoxic activity and proliferation of CD4^+^ and CD8^+^ T cells by reducing proinflammatory cytokine production (1). 1,25(OH)_2_D_3_ could enhance the anti-inflammatory effects of corticosteroids on monocytes and T cells *via* the induction of glucocorticoid receptor binding of steroids both *in vitro* and *in vivo* (22, 23). Combined treatment with steroid and 1,25(OH)_2_D_3_ may thus have therapeutic potential in patients with MS or NMOSD by decreasing the T-cell-mediated autoimmune processes. 1,25(OH)_2_D_3_ also exerts potent direct effects on B-cell responses, inhibiting their proliferation and differentiation into class-switched Bmem and plasma cells (6). However, there were no significant changes in the proportion of Breg and B cells in response to stimulation of PBMCs with 1,25(OH)_2_D_3_. Little is known about the effect of glucocorticoid on B cells. Dexamethasone could induce apoptosis of B cells at all stages (24), and especially immature B cells, and could stimulate T-cell-dependent immunoglobulin production by enhancing the differentiation of B cells into mature plasma cells without proliferation (25). In NMOSD, it was reported that the frequencies of mature Bmem increased after 2 weeks of high-dose steroid treatment (26). In the present study, the proportion of Bmem in PBMCs from NMOSD patients, which were initially reduced by stimulation with 1,25(OH)_2_D_3_, subsequently increased after stimulation with steroid, although the frequencies of Bmem did not differ between cells incubated with either 1,25(OH)_2_D_3_ or steroid alone. This may indicate that steroid-induced Bmem proliferation could be augmented by 1,25(OH)_2_D_3_ (22, 23). However, these findings were not observed in either HCs or MS patients; thus, further investigation is warranted.

It was particularly interesting that significant associations were observed between serum 25(OH)D_3_ and Bmem frequency in PBMCs stimulated with LPS+CD40L+CpG-ODN, with the correlation being negative in HCs and positive in NMOSD. In MS patients, the PBMC Bmem frequency was negatively correlated with serum 25(OH)D_3_ after preincubation with 1,25(OH)_2_D_3_. Clinical data regarding the relationship between Bmem and serum 25(OH)D_3_ in autoimmune diseases are scarce. In SLE, a significant negative association was identified between serum 25(OH)D_3_ and Bmem, but not total B cells or plasmablasts (27). MS patients with a low vitamin D status exhibited decreased cerebrospinal fluid levels of vitamin D and greater intrathecal accumulation of class-switched Bmem and antibody-secreting plasma cells (7). Since Bmem are considered to be a source of proinflammatory cytokines, which are responsible for pathogenic effects during autoimmune processes (28, 29), it seems to be contradictory that NMOSD patients with higher serum 25(OH)D_3_ would have higher Bmem frequencies. However, the immune balance represented by the Bmem/Breg ratio was not correlated with the vitamin D level in NMOSD. After PBMC stimulation, the proportion of Bmem was significantly decreased in NMOSD, unlike in MS. The significance of a lower proliferation of non-Bmem in NMOSD patients with higher serum 25(OH)D_3_ remains to be established. The present findings also suggest that the suppressive effect of vitamin D on Bmem is stronger in MS patients with higher serum 25(OH)D_3_. A recent meta-analysis suggested that vitamin D supplementation has a therapeutic role in the treatment of MS; however, there is uncertainty about the most appropriate dose and factors influencing the immune regulatory roles of 1,25(OH)_2_D_3_ (30). The therapeutic potential of vitamin D intake in patients with MS and NMOSD needs to be investigated further.

The biological activity of vitamin D is determined by the combination of levels of VDR expression and the activities of the metabolizing enzymes 1α-hydroxylase (CYP27B1) and 24-hydroxylase (CYP24A1) (1). 1,25(OH)_2_D_3_ acts mainly *via* VDR-mediated regulation of gene expression, and VDR transcription is induced by 1,25(OH)_2_D_3_ itself (31). IL-10 expression in activated B cells is enhanced by 1,25(OH)_2_D_3_ more than threefold, primarily through the recruitment of VDR to the promoter of IL-10 (32). However, the mRNA expression levels of *VDR* and *IL-10* in PBMCs were not significantly influenced by the presence of 1,25(OH)_2_D_3_ in the present study. This may be attributable to upregulated *VDR* expression by inflammatory signals offsetting the effects of 1,25(OH)_2_D_3_ on immune cells (3), or mRNA measurement times that were inappropriate for allowing the detection of any effect (33). In stimulated PBMCs, feedback regulation of vitamin D metabolism by 1,25(OH)_2_D_3_ was observed in HCs, and in patients with MS and NMOSD. The presence of CYP27B1 has been demonstrated in immune cells, enabling them to produce locally active 1,25(OH)_2_D_3_ from 25(OH)D_3_ (1). CYP24A1 expression is also induced by 1,25(OH)_2_D_3_, creating a self-regulatory feedback loop and enabling 1,25(OH)_2_D_3_ to fulfill its role in maintaining immune balance (31). It is noteworthy that in response to stimulation with 1,25(OH)_2_D_3_, *CYP24A1* mRNA expression was lower in MS patients with higher serum 25(OH)D_3_. This suggests that serum 25(OH)D_3_ is associated with impaired regulation of CYP24A1 activity. There are few previous reports on the associations between genetic polymorphisms in vitamin-D-regulated genes such as *CYP24A1*, vitamin D status, and MS risk (34, 35). Further studies with larger samples are needed to reveal the mechanism underlying the impaired regulation of vitamin D hydroxylation in MS.

This study had several limitations. The small number of samples in each study group must be considered when interpreting these data, which reduced the statistical power and may have introduced unintended bias. In addition, the enrolled patients were all in remission with heterogenous disease duration and had been taking immunosuppressive or disease-modifying drugs, which could affect immune cell function. Moreover, we did not obtain age-matched HCs compared with the NMOSD group, which could affect the composition and quality of the lymphocyte pool.

In conclusion, the findings of this study suggest that vitamin D plus steroid has a therapeutic benefit on T cells in MS and NMOSD, and that differential transcriptional activities of the CYP24A1 gene could exist that affect serum 25(OH)D_3_ in MS. In addition, vitamin D may have different inhibitory effects on Bmem that are dependent upon serum 25(OH)D_3_ in MS and NMOSD. The positive association between CD19^+^CD27^+^ B-cell frequency and serum 25(OH)D_3_ in NMOSD after immune-cell stimulation must also be further explored to establish whether vitamin D does have beneficial effects in this autoimmune disease, or if this was simply an accidental correlation. Further large-scale studies could help to elucidate the immunoregulatory mechanism of vitamin D supplementation in patients with MS and NMOSD.

## Data Availability Statement

The datasets presented in this study can be found in online repositories. The names of the repository/repositories and accession number(s) can be found in the article/**Supplementary Material**.

## Ethics Statement

The study was approved by the Institutional Review Board of Samsung Medical Center and written informed consents were obtained from all subjects.

## Author Contributions

Study concept and design: EBC and J-HM. Acquisition of data: EBC, JHS, SK, JK, JMS, BK, and J-HM. Analysis and interpretation of the data: EBC, BK, and J-HM. Draft and revision of the manuscript for content: EBC and J-HM. All authors contributed to the article and approved the submitted version.

## Funding

This research was supported by Basic Science Research Program through the National Research Foundation of Korea (NRF) funded by the Ministry of Education (2016R1D1A1B03934476).

## Conflict of Interest

The authors declare that the research was conducted in the absence of any commercial or financial relationships that could be construed as a potential conflict of interest.

## Publisher’s Note

All claims expressed in this article are solely those of the authors and do not necessarily represent those of their affiliated organizations, or those of the publisher, the editors and the reviewers. Any product that may be evaluated in this article, or claim that may be made by its manufacturer, is not guaranteed or endorsed by the publisher.

## Supplementary Material

Supplementary Figure 1 Changes in the proportion of lymphocytes in samples of PBMCs in response to stimulation with LPS+CD40L+CpG-ODN (nonspecific) in HCs and in patients with MS or NMOSD. A significant increase in total lymphocyte percentage was noted in the NMOSD group **(A)**. Relative B-cell proliferation compared with T cells was found in the MS and NMOSD groups **(B, C)**. The Bmem/Breg ratio was significantly reduced in HCs and patients with NMOSD, but not in those with MS **(D–F)**. Bmem, CD19^+^CD27^+^ memory B cell; Breg, CD19^+^CD24^+^CD38^+^ regulatory B cell; CD40L, CD40 ligand; CpG-ODN, cytosine phosphate guanosine oligodeoxynucleotides; HC, healthy control; LPS, lipopolysaccharide; MS, multiple sclerosis; NMOSD, neuromyelitis optica spectrum disorder; PBMC, peripheral blood mononuclear cell.
