# Supplementary figures and images for: Effects of Vitamin D and Dexamethasone on Lymphocyte Proportions and Their Associations With Serum Concentrations of 25-Hydroxyvitamin D3 In Vitro in Patients With Multiple Sclerosis or Neuromyelitis Optica Spectrum Disorder
Source: Front Immunol. 2021 Jul 29;12:677041. doi: 10.3389/fimmu.2021.677041 (PMC8358328; doi:10.3389/fimmu.2021.677041)

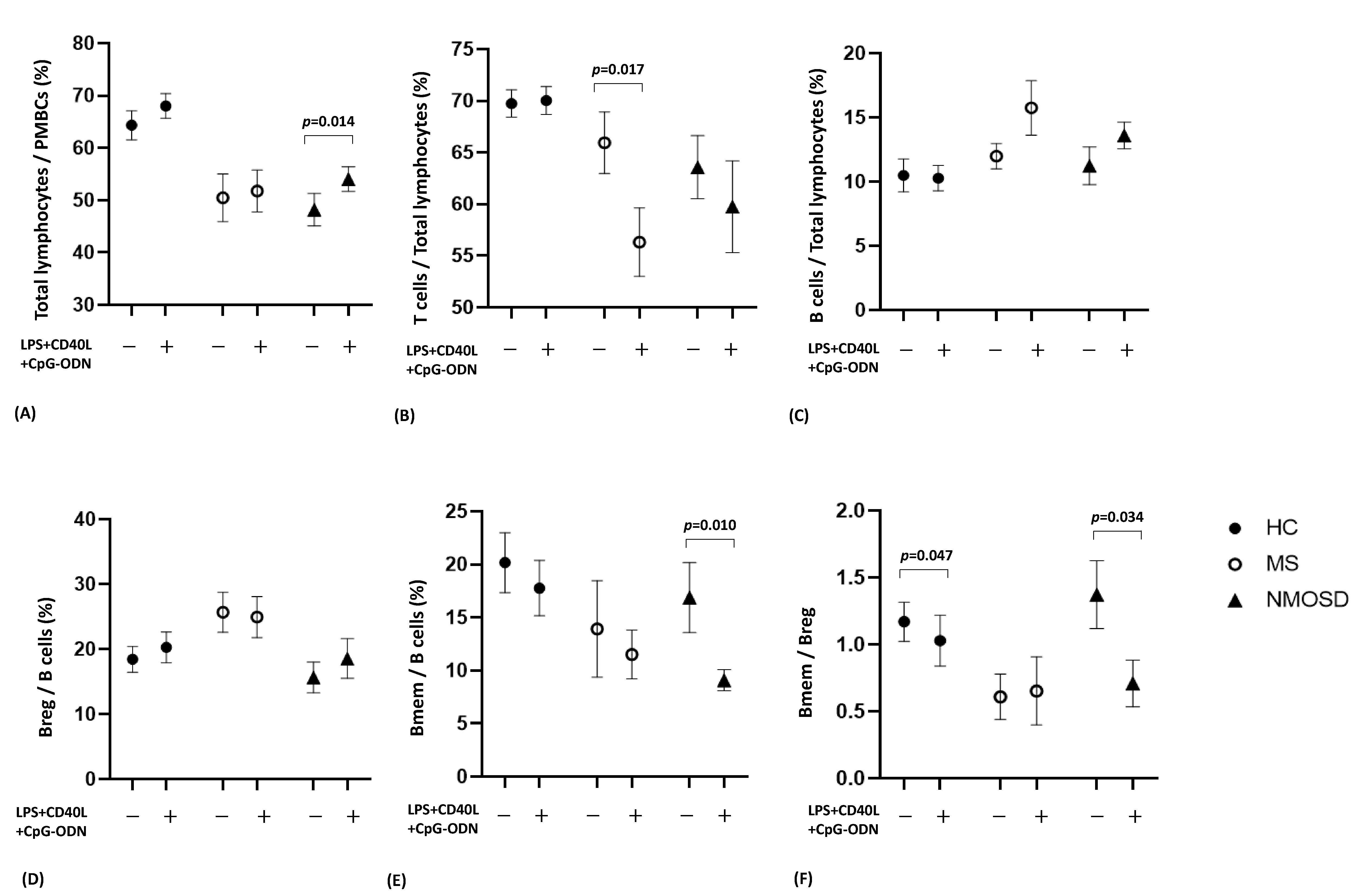

Supplement: Supplementary Figure 1 — Changes in the proportion of lymphocytes in samples of PBMCs in response to stimulation with LPS+CD40L+CpG-ODN (nonspecific) in HCs and in patients with MS or NMOSD. A significant increase in total lymphocyte percentage was noted in the NMOSD group (A). Relative B-cell proliferation compared with T cells was found in the MS and NMOSD groups (B, C). The Bmem/Breg ratio was significantly reduced in HCs and patients with NMOSD, but not in those with MS (D–F). Bmem, CD19+CD27+ memory B cell; Breg, CD19+CD24+CD38+ regulatory B cell; CD40L, CD40 ligand; CpG-ODN, cytosine phosphate guanosine oligodeoxynucleotides; HC, healthy control; LPS, lipopolysaccharide; MS, multiple sclerosis; NMOSD, neuromyelitis optica spectrum disorder; PBMC, peripheral blood mononuclear cell. [file Image_1.jpg]
