# Supplementary material for: Effects of Vitamin D and Dexamethasone on Lymphocyte Proportions and Their Associations With Serum Concentrations of 25-Hydroxyvitamin D3 In Vitro in Patients With Multiple Sclerosis or Neuromyelitis Optica Spectrum Disorder
Source: Front Immunol. 2021 Jul 29;12:677041. doi: 10.3389/fimmu.2021.677041 (PMC8358328; doi:10.3389/fimmu.2021.677041)
Supplement: Supplementary file 2 [file Table_1.docx]

**Table e-1** Demographics and serum concentrations of 25(OH)D_3_ of study subjects for gene expression

|  | HCs  (*N*=15) | MS  (*N*=11) | NMOSD  (*N*=13) | *p* value |
| --- | --- | --- | --- | --- |
| Age | 32.5$\pm$5.3 | 34.3$\pm$8.3 | 46.5$\pm$14.1 | 0.001^a^ |
| Female, *N* (%) | 8 (53.3) | 8 (72.7) | 12 (92.3) | 0.073 |
| Disease duration, months | N/A | 22.0 (4.0$-$52.0) | 25.0 (12.0$-$96.65) | 0.469 |
| Serum 25(OH)D_3_, mg/dl | 17.2$\pm$4.8 (*N*=14) | 21.7$\pm$10.4 (*N*=10) | 32.2$\pm$16.7 | 0.010^a^ |
| On taking 1,25(OH)_2_D_3_, *N* (%) | 0 | 3/10 (30.0) | 4 (30.8) | 0.068 |
| ARR | N/A | 0.7 (0.33$-$4.0) | 0.5 (0.1$-$1.1) | 0.369 |
| EDSS | N/A | 1.0 (0.0$-$2.5) | 3.0 (1.25$-$3.25) | 0.136 |
| Use of drugs, *N* (%) | N/A | 9 (81.8)^b^ | 11 (84.6)^c^ | 0.855 |

N, number; ARR, annualized relapse rate; EDSS, Expanded Disability Status Scale; HCs, healthy controls; MS, multiple sclerosis; NMOSD, neuromyelitis optica spectrum disorder; Values are presented as either mean$\pm$SD or median (IQR) unless otherwise indicated.

^a^significant between NMOSD and HCs or MS

^b^interferon β-1b (n=5), interferon β-1a (n=3), and teriflunomide (n=1)

^c^azathioprine (n=6), mycophenolate mofetil (n=3), hydroxychloroquine (n=1), and methotrexate (n=1)
